# Targeted Whole Exome Sequencing in Children With Early-Onset Epilepsy: Parent Experiences

**DOI:** 10.1177/08830738221113901

**Published:** 2022-08-03

**Authors:** Armaghan Alam, Maksim Parfyonov, Camille Y. Huang, Inderpal Gill, Mary B. Connolly, Judy Illes

**Affiliations:** 1 Neuroethics Canada, Division of Neurology, Department of Medicine, 8166 University of British Columbia, Vancouver, British Columbia, Canada; 2 Faculty of Medicine, 8166 University of British Columbia, Vancouver, British Columbia, Canada; 3 Department of Pediatrics, Division of Neurology, 37210 BC Children’s Hospital, Vancouver, British Columbia, Canada

**Keywords:** children, epilepsy, ethics, genetics, mutation, next-generation sequencing, outcome, seizures, and treatment

## Abstract

This study investigated the experiences of 25 caregivers of children with early-onset, treatment-resistant epilepsy who pursued whole exome sequencing to determine the impact of the test results on their child’s treatment. Caregivers who consented to be recontacted were recruited from a previous study investigating the diagnostic yield of whole exome sequencing. A semistructured interview addressed questions based on one of 2 study phases. The first phase discussed the decision-making process for genetic testing (15 interviews), which revealed 4 major themes: (1) prognosis, (2) engagement, (3) concerns, and (4) autonomy. The second phase discussed the impact of genetic testing on treatment (10 interviews), which revealed 3 major themes: (1) testing features, (2) emotional impact, and (3) treatment outcomes. Overall, parents pursued genetic testing to obtain a clear prognosis, inform treatment decisions, engage with other families, and exercise autonomy. Caregivers felt that early testing is warranted to inform their child’s diagnostic odyssey.

## Introduction

Epilepsy is one of most common pediatric neurologic conditions affecting approximately 0.5% to 1.0% of children worldwide.^1^ To date, more than 900 genes have been implicated in epilepsy,^2^ and an estimated 70% to 80% of epilepsies may have a genetic basis.^3^ Identification of a genetic diagnosis may enable clinicians to provide individualized genetic counseling on prognosis and developmental outcomes, recurrence risk, and enable targeted treatment. Accordingly, next generation sequencing technology, including whole exome sequencing and epilepsy gene panels, play a major role in the diagnosis and management of the disease.^4,8^

Although several studies have explored parent perceptions of genetic testing in other pediatric conditions^9,13^ and the peer-reviewed literature have delineated the treatment impacts and the ethical and psychosocial considerations of genetic testing in epilepsy,^14,18^ there is a lack of data on parent attitudes toward next generation sequencing in pediatric epilepsy, and how these attitudes inform decision making about testing and treatment. In the present study, we explored the perceptions and experiences of caregivers of children with early-onset, treatment-resistant epilepsy in a cohort in British Columbia, Canada,^14^ who underwent targeted whole exome sequencing, and the impact of the genetic test results on their child’s treatment and care.

## Materials and Methods

### Participants

Parents of children with early-onset epilepsy were recruited from a pool of participants in a study investigating the diagnostic yield of targeted whole exome sequencing conducted at BC Children’s Hospital, British Columbia, Canada.^14^ Children in the original study had onset of seizures at ≤5 years of age of unclear cause after extensive investigations. The parents of 21 children provided informed consent to participate in the present study, and all had provided consent for recontact in the original study (BC Children's Hospital and University of British Columbia Ethics Board H18-02783). Both parents participated for 4 of the children; therefore, a total of 25 parents of 21 children were interviewed. Where 2 parents participated, they were interviewed separately and treated as separate units for analysis. Pre- and posttest genetic counseling was provided for each patient or family.

### Setting

The study had 2 phases: (1) decision making for genetic testing (15 interviews) and (2) impact of genetic testing on treatment choices (10 interviews). In the original study, parents received either a positive result associated with a known epilepsy-causing gene, a negative result in which no known epilepsy gene was identified, or a variant of uncertain significance (VUS).

Of the 15 interviews in phase 1, 7 parents had received a positive result, 6 a negative result, and 2 a VUS. In phase 2, purposive sampling was used to select parents who had received a positive result as they were most likely to have experienced a change to their child’s treatment regimen, so of the 10 interviews, 7 parents received a positive result and 3 received a negative result. All interviews were conducted between 1.46 and 5.41 years after parents received the genetic test results.

### Interviews

A summary of the interview questions is included in Table 1. Interview guides for both phase 1 and 2 were semistructured and utilized open-ended questions with probes to elicit discussion. The guide for the phase 1 work was developed based on the peer-reviewed literature of other studies of parent perceptions and genetic testing.^19,22^ It was then vetted and refined based on feedback from the research team and the clinicians providing care to the families. This interview guide focused on the motivations and reservations parents had when pursuing genetic testing for their child, as well as the general impact of these results on the family. The second interview guide was developed after 15 phase 1 interviews were completed to further explore the perceived impact of genetic testing results on treatment. Purposive sampling of parents with positive results was used for the phase 2 work as they were most likely to have experienced a change to their child’s treatment. Accordingly, parents were recruited from among the 59 original study participants who received a pathogenic variant.^14^ Given the small sample size, it was difficult to select for the parents of the 23 patients within this group who had reported changes to their clinical management in the original study.^14^ In the end, only 2 were consented and interviewed.

**Table 1.** Summary of Interview Questions.

Question	Probes
Questions Common to Both Phases	
What is your age?	*21-40, 41-60, 61-80 y/o*
With what gender do you identify?	*Female, male, other, or prefer not to say*
What is your highest educational degree?	*Less than high school, high school, university, postgraduate*
How many members are there in your immediate family (eg, living at home)?	
Phase 1: Decision-Making for Genetic Testing	
Do you feel like you had all the information you needed about genetic testing for your child in order to make your decision?	Please tell me what information was most helpful? Please tell me what you would have liked more information about?
What motivated you to pursue genetic testing for your child?	Some prompts included: *learning what caused the epilepsy, being better able to care and advocate for child through targeted treatment, reducing guilt and blame, reproductive decision making, increasing sense of control, and connecting with families with the same diagnosis*
Did you have any concerns or doubts about the genetic test before agreeing to do it?	Some prompts included: *procedure, inability to understand results, continued therapeutic uncertainty, and receiving a positive result and the impact that would have on reproductive decision making (future children for yourself and your child)*
Do you feel the results are meaningful?	
Did the results of the genetic test change the course of treatment for your child? Please elaborate.	
Did you have any concerns after you received the results?	Some prompts included: *concerns about epilepsy in future children, continued uncertainty, blame and guilt, increased stigma, insurance discrimination, and self-imposed limitations on child’s life goals*
With whom did you share/discuss the results (family members, close friends, etc)? Please elaborate.	Did you connect with any other families online or research groups studying your child’s genetic change? Could you explain why or why not?
Were the results otherwise helpful to you? Please explain.	
Phase 2: Impact of Genetic Testing on Treatment Choices	
How did the results of the genetic test change the course of treatment for your child (eg, medical, surgical, other)?	Were there any treatments suggested based on your results? Were there any treatments contraindicated based on your results? Did your child undergo any surgical procedures for epilepsy after receiving genetic testing? How much did the genetic results inform that decision?
How much did you expect treatment options to change based on genetic testing? Were those expectations met?	Did you feel more or less motivated to pursue treatment after receiving your genetic test results? Why? Did you feel like you were sufficiently explained how genetic test results may or may not impact your child’s treatment? Why?
Did your treatment priorities change after receiving the genetic testing results?	Some prompts included: e*fficacy, safety, adverse events, intensity of follow-up, requires traveling to treatment center, cost, clear information about procedure is available, invasiveness, or a new intervention*
Do you feel the results of your genetic test changed your views on or values about different treatment interventions, such as drug combinations, resective surgery, deep brain stimulation, vagal nerve stimulation? If yes, how? If no, why not?	Do you feel more or less likely to consider these types of interventions (specifically invasive brain procedures)?

The one-on-one interviews were conducted by authors A.A. or M.P. virtually on Zoom because of the COVID-19 pandemic. They ranged from 15 to 30 minutes. All sessions were audio recorded, encrypted, and then uploaded to a secure institutional server. Phase 1 interviews were conducted from May 2020 to October 2020. Phase 2 interviews were conducted between February 2021 and June 2021.

### Data Analysis

Interview recordings were professionally transcribed and imported into NVivo software (QSR 12) for analysis using the constant comparative approach.^23^ Two members of the study team (AA, MP) trained in qualitative methods, independently reviewed the transcripts, and coded them to identify salient themes. Interrater reliability was established in 15% of the transcripts by resolving inconsistencies in coding (Cohen kappa ≥ 0.8).^24^

A codebook was constructed before the interviews, with additional labels incorporated as they emerged through inductive and deductive analysis of the transcripts. Through iterative analysis, these labels were then coalesced to form subthemes and then finally themes. Illustrative quotes are used here to elaborate on salient thematic points, and ellipses applied for clarity and readability.

Results were visualized quantitatively into a pedigree structure based on data from the interviews: major thematic branches (topmost level), major themes, and minor themes following the approach by Hrincu et al.^21^ Major themes constituted the top 50% of most frequently coded topics in each thematic branch. Minor themes represent relative quantitative status and provide qualitative depth and insight. Themes were ranked by frequency of references (n) in the respective phase of the study. Subthemes were calculated by using the frequency that a theme was discussed (n) across all interviews in that specific phase as the denominator and the frequency a specific subtheme was discussed within that given theme as the numerator.

In addition to qualitative data analysis from the interviews, patient’s charts were reviewed to characterize treatment impact following genetic testing. Changes to treatment course or medications in the six months preceding the time when genetic results were shared with the family were documented to establish a baseline and to record any changes that could not be attributed to the genetic findings. The six months following were used to record any significant treatment or medication changes. All such changes were reviewed by the clinicians involved in this study to determine whether they resulted directly from the genetic testing. Treatment impact was also documented and cross-referenced with the original study by Demos et al^14^ and are shown in Table 2.

**Table 2.** Demographics of the Children of Participants.

Study ID	Current age	Gender	Age of onset	Diagnosis	Age at genetic testing	Result	Gene finding	Treatment impact^a^
EPGEN026	11	F	43 mo	EE	4 y 10 mo	Negative	N/A	
EPGEN029	19	M	3 mo	Focal epilepsy	12 y 8 mo	Negative	N/A	
EPGEN037	12	F	62 mo	Focal epilepsy	5 y 7 mo	Negative	N/A	
EPGEN087	8	F	11 mo	Unclassified	2 y	Negative	N/A	
EPGEN101	16	F	18 mo	EE	10 y 2 mo	Negative	N/A	
EPGEN103	10	F	52 mo	Unclassified	4 y 8 mo	Negative	N/A	
EPGEN176	8	F	27 mo	Focal epilepsy	2 y 6 mo	Negative	N/A	
EPGEN186	9	M	35 mo	Focal epilepsy	3 y 2 mo	Negative	N/A	
EPGEN030	13	M	42 mo	Generalized epilepsy	6 y 8 mo	VUS	*DIP2B*	VNS implanted a few mo before genetic results were shared. VNS resulted in complete seizure control
EPGEN052	14	F	3 mo	Focal epilepsy	7 y 5 mo	VUS	*WDFY3*	
EPGEN067	22	M	19 mo	LGS	15 y 11 mo	Positive	*SCN5A*	No impact on epilepsy management. Found to be inherited and familial screening for cardiac risk performed
EPGEN072	25	M	29 mo	Focal epilepsy	18 y 11 mo	Positive	*TCF20*	
EPGEN073	23	F	29 mo	EE	16 y 6 mo	Positive	*YWHAG*	
EPGEN077	16	F	2 mo	West, LGS	8 y 8 mo	Positive	*CDKL5*	
EPGEN106	23	F	10 mo	Focal epilepsy	16 y 9 mo	Positive	*STXBP1*	
EPGEN130	25	F	2 mo	West; focal epilepsy	19 y 1 mo	Positive	*DEPDC5*	
EPGEN165	8	M	23 mo	Dravet syndrome	2 y 3 mo	Positive	*SCN1A*	No changes to ASMs, but identified medications contraindicated for children with *SCN1A* pathogenic variants
EPGEN197	6	M	6 mo	Dravet syndrome	7 mo	Positive	*SCN1A*	No changes to ASMs, but identified medications contraindicated for children with *SCN1A* pathogenic variants
EPGEN208	11	F	11 mo	PCDH19-related EE	7 y 3 mo	Positive	*PCDH19*	Prednisone (20 mg at onset of cluster of seizures) started, known to be effective in some patients with *PCDH19­*, not continued because of lack of efficacy
EPGEN230	15	F	5 mo	West, LGS	10 y 11 mo	Positive	*SCN8A*	Sodium channel blocker started, not continued because of adverse effects
EPGEN248	28	F	38 mo	Unclassified	25 y 4 mo	Positive	*SCN1A*	No changes to ASMs, but identified medications contraindicated for children with *SCN1A* pathogenic variants

Abbreviations: ASMs, antiseizure medications; CAE, childhood absence epilepsy; EE, unspecified epileptic encephalopathy; GEFS+, genetic epilepsy with febrile seizures plus; LGS, Lennox Gastaut syndrome; M, Months; VUS, variant of uncertain significance; VNS, vagus nerve stimulation; West, West Syndrome.

^a^ Patient’s charts were reviewed to determine treatment impact. We documented changes to treatment course or medications in the 6 months preceding when genetic results were shared with the family. The 6 months preceding were used to establish a baseline and to record any changes in treatment plan that could not be attributed to the genetic findings. The 6 months following were used to record any significant treatment or medication changes. If there were changes, these were reviewed by the clinicians involved in this study to determine if these changes were a direct result of the genetic testing results. The resulting treatment impact was also documented in the original study by Demos et al.

## Results

### Participants

The parents interviewed in this study had children with a mean age of onset of epilepsy at 22.38 ± 17.84 months and a mean age at time of genetic testing of 9.61 ± 6.86 years (Table 2). Table 2 summarizes the demographics of children of the study participants. Mean duration of the interviews was 16 minutes for both study phases. The average time between when the families received their genetic testing results and when they were interviewed for this study was 4.22 years.

In phase 1, of the 15 parents interviewed, 7 received a positive result, 6 received a negative result, and 2 received a VUS. In phase 2, of the 10 parents interviewed, 7 received a positive result and 3 received a negative result (Table 1). Eighty percent of parents interviewed were female, and the mean age of the parents was 47 ± 7.53 years. All but 6 participants (76%) had at least college- or university-level education. Table 3 summarizes the demographics of the study participants.

**Table 3.** Demographics of Parents Interviewed.

Study ID parent	Age	Gender	Education background	Number of immediate family members	Phase of interview	Result
EPGEN026	39	F	Bachelor’s or equivalent	4	1	Negative
EPGEN030	41	M	Secondary education	4	1	VUS
EPGEN037	44	F	Doctoral or equivalent	4	1	Negative
EPGEN052	50	F	Bachelor’s or equivalent	5	1	VUS
EPGEN067	53	F	Bachelor’s or equivalent	3	1	Positive
EPGEN077	42	F	Bachelor’s or equivalent	10	1	Positive
EPGEN087	40	M	Bachelor’s or equivalent	4	1	Negative
EPGEN087	39	F	Bachelor’s or equivalent	4	1	Negative
EPGEN101	46	M	Bachelor’s or equivalent	5	1	Negative
EPGEN176	41	F	Bachelor’s or equivalent	5	1	Negative
EPGEN186	37	F	Bachelor’s or equivalent	4	1	Negative
EPGEN230	47	M	Secondary education	4	1	Positive
EPGEN230	50	F	Secondary education	4	1	Positive
EPGEN248	61	M	Bachelor’s or equivalent	3	1	Positive
EPGEN248	63	F	Secondary education	3	1	Positive
EPGEN029	59	F	Secondary education	4	2	Negative
EPGEN072	52	F	Bachelor’s or equivalent	5	2	Positive
EPGEN073	51	F	Bachelor’s or equivalent	4	2	Positive
EPGEN101	47	F	Master’s or equivalent	5	2	Negative
EPGEN103	36	F	Bachelor’s or equivalent	4	2	Negative
EPGEN106	52	F	Bachelor’s or equivalent	3	2	Positive
EPGEN130	55	F	Secondary education	4	2	Positive
EPGEN165	42	F	Bachelor’s or equivalent	4	2	Positive
EPGEN197	40	F	Master’s or equivalent	4	2	Positive
EPGEN208	48	F	Master’s or equivalent	4	2	Positive

Abbreviation: VUS, variant of uncertain significance.

### Phase I: Decision Making for Genetic Testing

**Figure 1.**
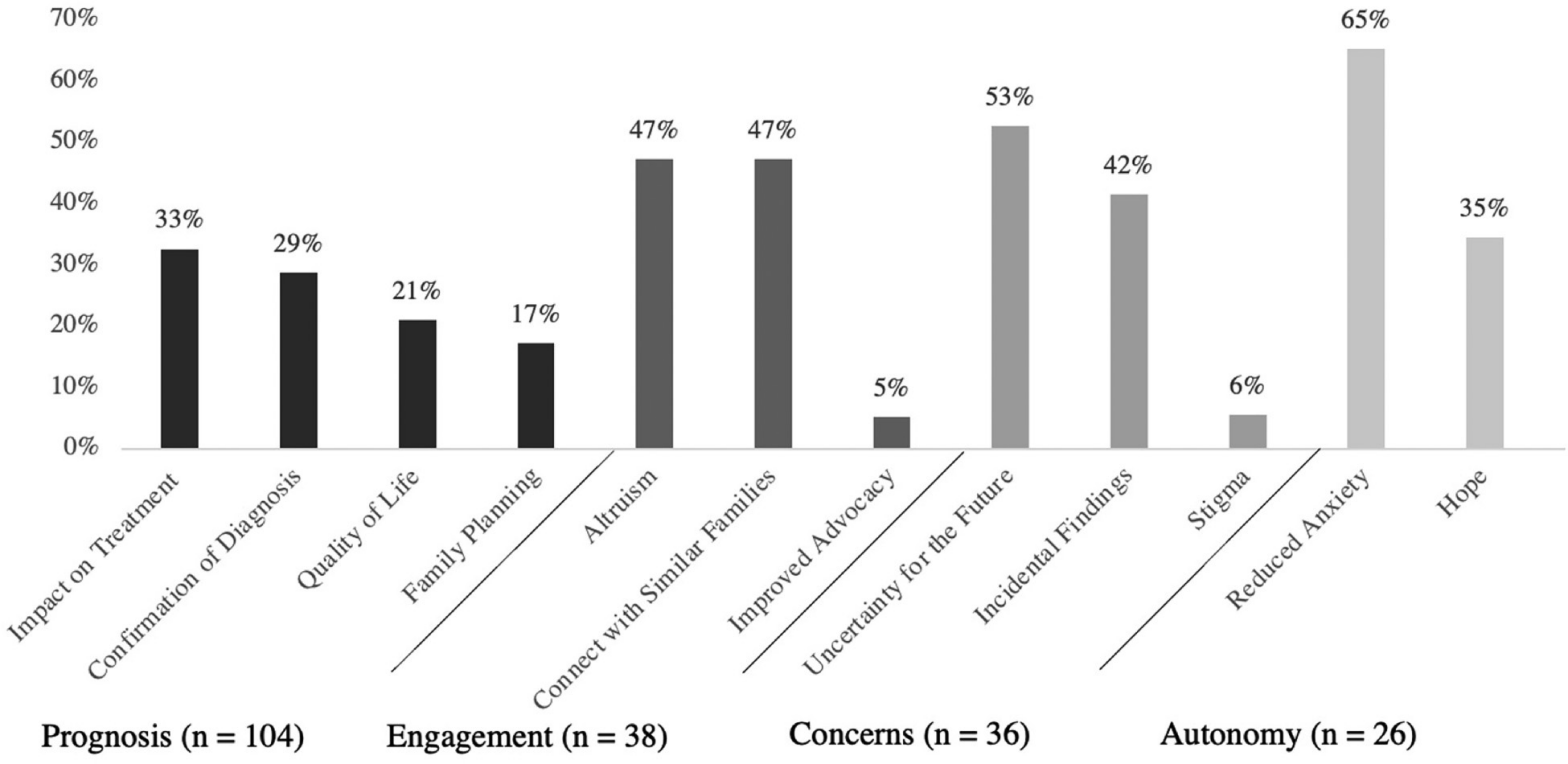
Major themes identified in phase 1: decision making for genetic testing (n = frequency of themes).

The first phase of interviews focused on decision making for genetic testing, which revealed 4 major themes: (1) prognosis, (2) engagement, (3) concerns, and (4) autonomy (Figure 1).

#### Prognosis

Parents across all interviews identified a desire to better understand their child’s long-term prognosis as a key motivator to pursue whole-exome sequencing. Even among parents who received negative results, parents were universally optimistic that genetic testing would allow for novel, targeted treatment options:

I think they were helpful just to give us an idea of what was causing her seizures, and that there was a possibility of different treatments based on where her seizures were coming from, and that [certain treatments] might be more useful than others. (EPGEN248—F, phase 1, positive)

Similarly, many parents felt that identifying an etiology for their child’s epilepsy even in the absence of novel treatment options would provide closure and a definite cause for their child’s condition. For those who received negative or VUS results, parents still felt hopeful that future analysis could uncover additional causative variants. Furthermore, themes of quality of life and family planning centered on the hope that these results would bring their child a step closer to being seizure-free or allow them to have more children without the risk of hereditary epilepsy in their children or grandchildren.

#### Engagement

Under the theme of engagement, parents expressed their desire to engage with other families either through research, peer support, or advocacy. Many parents suggested that altruism was a leading reason to pursue genetic testing:

[I] have a science background so I know all about genetics and there’s not much I guess we can do for . . . her genetics but just to figure out for other people. (EPGEN087—F, phase 1, negative)

Similarly, parents had a significant desire to connect with other families to share their experiences and receive peer support in dealing with difficult diagnoses. For example:

[I remember thinking] Wow there's other people out there with the same diagnosis although [with] huge variations. . . . Some are walking and talking, . . . and others are you know even worse than [my daughter]. But you felt like you're not alone, right? (EPGEN230—M, phase 1, positive)

Parents who received a positive result also found that it helped legitimize their child’s condition and allowed for improved advocacy on their behalf. One parent stated that health care providers responded to her concerns in the emergency department with more attention and candor when she stated that her child had a mutation causing their seizures when compared to visits before she had received a diagnosis.

#### Concerns

Another theme that was identified during the interview process were concerns about the future and about incidental findings that parents associated with genetic testing. Uncertainty about the future was particularly prevalent among the parents who received negative and VUS results. These parents reported frustration and ambiguity with determining the appropriate next step in their child’s care in an already lengthy therapeutic journey, while still maintaining hope that new advances in epilepsy would uncover an etiology at some point in the future. For some parents, disclosure of incidental findings was beneficial and necessary, for others less so. In one interview, the parent stated that they discovered that their child had G6PD deficiency through the genetic testing process—a finding that allowed them to provide better care and avoid dangerous complications in a child that already had significant existing medical conditions.

#### Autonomy

The final theme identified in the first phase of interviews was autonomy. Parents expressed that pursuing genetic testing provided them with an increased sensation of control and a reduced sensation of guilt and anxiety over their child’s condition. This was demonstrated by the subthemes of hope and reduced anxiety respectively. The largest theme in this branch was a reduced sense of anxiety which was exclusively found in the positive result interviews:

As a parent you're always wondering - you think, what did I do? Did I eat something, or did I drink something? . . . So for 11 or 12 years you're always second-guessing every little thing you did [ . . . and] it's like this huge weight off your shoulders. (EPGEN230—M, phase 1, positive)

The father of participant EPGEN230, interviewed independently, noted:

Children that have epilepsy . . . should immediately go in for genetic testing so if they do have something like this, they can properly be medicated. It’s unbelievable how much guilt my wife harbored for years, and years, and years. And if she had only known you know, within the . . . year after [my daughter] was diagnosed if she just had known then you know her mindset would've changed immensely. And her guilt, I mean she probably lost a couple years off her life with the amount of guilt she harbored. . . . Even as a husband I couldn't say . . . you never caused this. (EPGEN230—F, phase 1, positive)

Parents also reported feeling hopeful for the future while pursuing genetic testing regardless of the results they received. One parent expressed that being involved in the study reassured them that epilepsy research was an evolving field that was continuing to uncover new innovations in diagnosis and treatment.

### Phase 2: Impact of Genetic Testing on Treatment Choices

**Figure 2.**
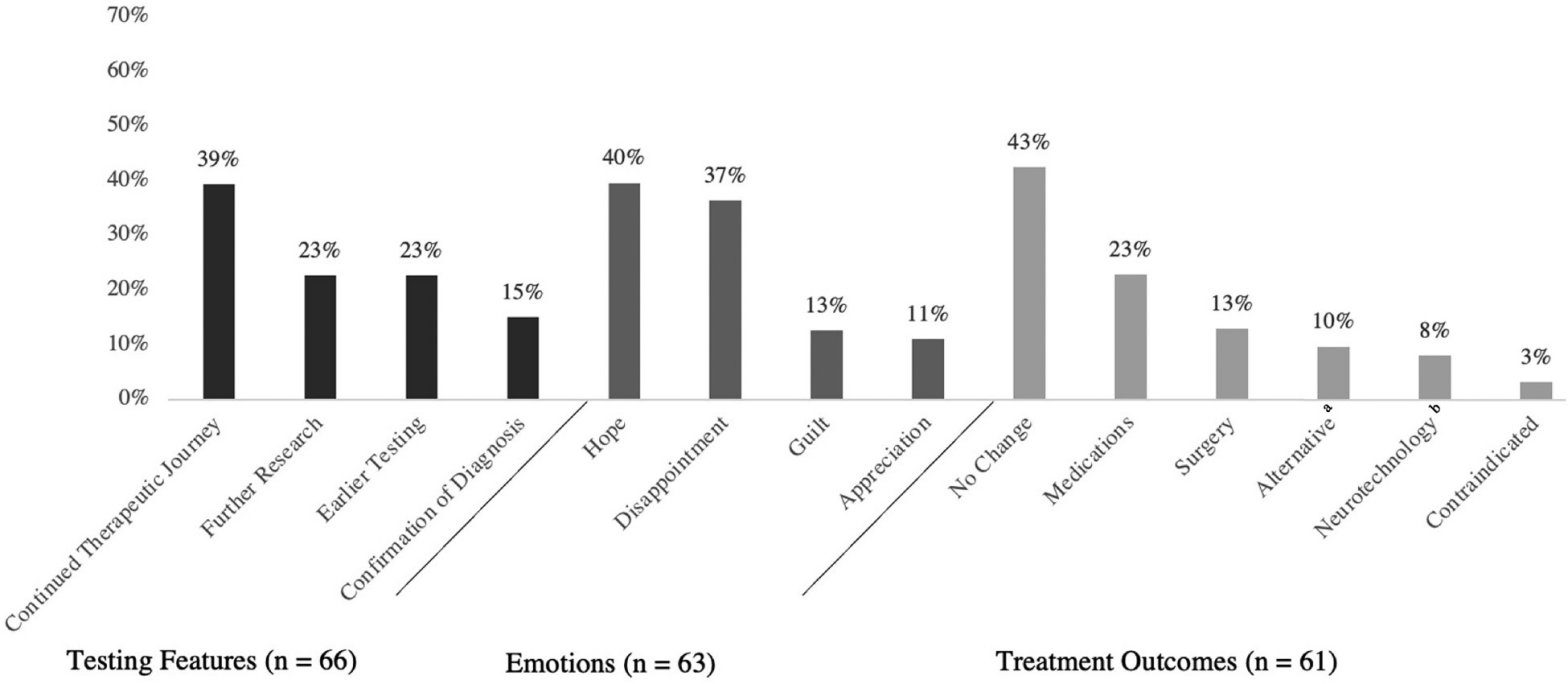
Major themes identified in phase 2: impact of genetic testing on treatment choices (n = frequency of themes).

The second phase of interviews focused on the impact of genetic testing on treatment choices that revealed 3 major themes: (1) testing features, (2) emotional impact, and (3) treatment outcomes (Figure 2).

#### Testing features

Testing features were attributes of the genetic testing process that parents felt should be addressed to improve the utility of genetic testing in providing targeted treatment options. An important subtheme identified here was the continued therapeutic journey:

After the test, [ . . . we knew] which medication is bad for him. We still need to try to find the right medication. (EPGEN165, phase 2, positive)

Some parents expected that identifying a causative gene mutation would lead to immediate changes to treatment, when only one of the children included in this study had a change made to their treatment regimen. Many of the parents reported facing continued uncertainty in their therapeutic journey and, accordingly, many of the parents interviewed reported participating in additional research studies.

Some parents identified the importance of early testing. Some parents stated that earlier testing would have allowed them to connect with peer support communities sooner, or that it would have eased long-term anxiety. In the context of treatment outcomes, some parents felt that earlier testing would have provided additional treatment options due to an earlier diagnosis:

Maybe if genetic testing had been available that many years before, there would [ . . . be more drugs that] worked for this type of seizure a little more specifically and just a little more grouping of similar patients (EPGEN073, phase 2, positive)

One parent further described feeling frustrated after receiving a positive result and joining a peer support group in which they became aware that children in other countries had received testing 10 years earlier and a diagnosis sooner.

#### Emotions

Parents described a variety of emotions when learning the results of genetic testing. Disappointment was a common response, particularly with a negative or VUS result:

It was kind of frustrating. Like, what’s the point of doing the testing? . . . Inconclusive in my mind means I don’t know so if you don’t know, then you find out. You keep going. You figure out why you don’t know, and so I was kind of annoyed and frustrated that inconclusive just seemed like a valid answer. (EPGEN103, phase 2, negative)

Another parent specifically felt disappointed that the genetic testing did not change treatment options:

There is something really valuable to having a name for it. I think there still would have been the exasperation, disappointment of, what? You don’t know how to treat it? (EPGEN208, phase 2, positive)

Similarly, hope was a subtheme that was present in many interviews. Most parents expressed hope for this research leading to new treatment options in the future, but one expressed a loss of hope following the genetic testing results:

Prior to receiving the diagnosis, we had some hope that it was something he might grow out of, or something that might respond really well to medical treatment, and so there was some, I guess, some hope, prior to receiving the diagnosis and the genetic testing results, that this might go away, eventually, or he may grow out of it, or it might be really well controlled. And then I guess when we received that—those results, then that kind of changed. (EPGEN97, phase 2, positive)

Appreciation was another subtheme expressed by parents. One shared that despite no new available treatment to her child, she was still grateful for the result:

If there was a treatment we would have been thrilled, but it was more about what caused it and the fact that we found that out was great too. (EPGEN130, phase 2, positive)

Another parent stated that she appreciated receiving the genetic test results at an earlier stage of her son’s condition:

I think overall we felt really fortunate that we were able to access the genetic testing when we did. I don’t think—going through it, I don’t think we really realized that—what the alternative would have been. Because, like I say, it all just kind of happened in the first few months after he started to have seizures, and so I feel lucky now that we didn’t waste, like, four or five years using the wrong medications and having worse outcomes than we’ve had. (EPGEN197, phase 2, positive)

#### Treatment outcomes

The final theme identified pertained to perceptions of different treatment outcomes after receiving genetic testing results: parents most frequently discussed *no change to treatment*, medications, and surgical management. Alternative treatments were also mentioned by a few parents that included any approach that could be pursued independently without clinician oversight and was not a conventional treatment modality. For example, one parent discussed the following:

After knowing that [my daughter] had a gene mutation . . . we kind of said, OK, well, we don’t have anything, you know, scientific; we’re not doctors. We’re just going to attempt things and see if anything works. . . . CBD does work with the mind. . . . it just caused us to now dig in and try whatever was out there. (EPGEN130, phase 2, positive)

Neurotechnology was another subtheme that was discussed. This included deep brain stimulation, vagus nerve stimulation, responsive neurostimulation, MRI-guided laser interstitial thermal therapy, and stereotactic radiosurgery. One parent stated:

I suspect that the presentation of what’s going on for her may—with PCDH19, make it that it’s less likely that she would be a good candidate for [VNS]. (EPGEN208, phase 2, positive)

### Overall Summary of Treatment Impact

On review of the patients’ charts, only 2 children in the cohort experienced a change to their treatment regimen as a direct result of genetic diagnosis. In a girl with a *PCDH19* pathogenic variant, a trial of prednisone during seizures clusters was introduced. However, it resulted in seizures that were longer and more intense and was therefore discontinued. Similarly, in a girl with an *SCN8A* pathogenic variant, a trial of sodium channel blockers was started but discontinued due to difficulties experienced with sleep patterns on the medication. A pathogenic variant in *SCN1A* in 3 other children informed medications contraindicated in this condition. One child was found to have an *SCN5A* pathogenic variant that was maternally inherited, which allowed familial screening for cardiac risk to be performed. There was no change to the child’s management plan, however. In one child, a vagus nerve stimulation device had been implanted in August 2015, a few months before the family received the genetic results, which was VUS. There were no changes to the treatment plan following the genetic testing results, and the child continued to have upwards of 100 five- to eight-second seizures per day. Unrelated to the genetic testing results, vagus nerve stimulation settings were switched to rapid cycling in October 2016, which significantly reduced seizures from 100 to 2 or 3 per day. Overall, none of the parents interviewed reported changes in surgical management or options for neuromodulatory treatment of their child’s epilepsy based on genetic results.

## Discussion

We used qualitative semistructured interviews to assess the motivations, concerns, and experiences of caregivers as they navigate the process of genetic testing for their child’s epilepsy and how receiving genetic testing results impacts treatment and care. The findings demonstrate that caregivers balance a myriad of perceived risks and benefits in their decision to pursue genetic testing. Although all participants reported that genetic testing was necessary, several had concerns around the implications of continuing uncertainty with negative or VUS results and incidental findings. Parents were motivated by the search for a definitive diagnosis, prognosis, and novel treatment avenues. They sought to connect with other families and obtain support as a result of a genetic diagnosis.

In terms of treatment choices, parents felt that the continued therapeutic journey and the importance of earlier testing should be addressed to improve the utility of genetic testing to provide targeted treatment options. Emotions such as hope, disappointment, and appreciation were common and should be carefully considered in the context of providing genetic counseling following the distribution of test results to families. Several families indicated frustration and disappointment around obtaining variants of uncertain significance, underscoring the importance of pretesting genetic counseling and informed consent to educate families about the range of possible outcomes. Altogether, the majority of parents interviewed believed that the benefits of receiving a genetic result outweighed any perceived risks or concerns, and that earlier testing may have resulted in better outcomes for their child.

### Comparison With Similar Studies

Previous qualitative studies have found that the primary concerns of parents are choice and personal utility, referring to potential risks for their child such as stigmatization, employment/insurance discrimination, loss of privacy, and restrictions on reproductive decision making.^25^ In particular, perceived and personal utility is becoming an increasingly well-established construct in genomics to discuss the personal, psychological, and social value of a genetic test result to the patient and their family, beyond clinical utility.^26,27^ Kohler et al^27^ defined this construct through a systematic review of the literature, which identified 15 distinct elements organized into 4 domains: affective, cognitive, behavioral, and social outcomes. In 2021, Hayeems et al^28^ further defined Kohler’s work in another systematic review in the context of parents/caregivers, which identified the same elements in addition to a new medical management domain. Of note, the present study, developed independently of Kohler’s and Hayeem’s work around the construct of personal utility, identified many of the same themes such as altruism, confirmation of diagnosis, and family planning, which map to their elements of “feeling good for helping others,” “knowledge of condition,” and “reproductive autonomy,” respectively.^27,28^ Phase 2 of the study also expands on the new domain of medical management identified by Hayeem et al^28^ by further exploring how parents/caregivers perceive genetic test results to alter treatment.

Collectively, the perceived benefits of genetic testing in this study echo previous work: receiving genetic results provides parents with important benefits such as closure, advocacy for the specific needs of their child, decreased guilt and anxiety, and in select cases, novel treatment options.^29,30^ Similar to the parents interviewed by Anderson et al,^31^ parents in the present study identified instrumental value in next generation sequencing technology, given the information it provides about their child’s condition, improved treatment, family planning purposes, and contribution to science.

The improved coverage and resolution of next generation sequencing techniques raise novel concerns related to the identification of variants of uncertain significance (VUS) and the identification of variants unrelated to the primary phenotype (incidental, or secondary variants). Despite this concern, previous research has indicated that most parents wish to know all results from genetic testing including primary variants, secondary variants, carrier status, adult-onset, and nonmedically actionable disease.^22,25^ Motivating factors include preparing for the future, the possibility of later developments in medical prevention and treatment, and the right to information. This was also the case here. The right to information and a personal approach in disclosing VUS and secondary variants is a common link between several studies,^30,32,33^ accenting parent to family and expert to parent communication as well as genetic counseling services to support the return of unexpected findings. Although parents have reported concerns around associated risks such as insurance discrimination and psychological distress with secondary variant disclosure,^31^ this was not indicated in our interviews.

Finally, a recent study by Jeffrey et al provides an opportunity to further contextualize the present one. Jeffrey et al evaluated parent experiences after receiving a positive genetic testing result for children with developmental and epileptic encephalopathy that identified 3 key themes: “Importance of the label,” “Relief to end the diagnostic journey,” and “Factors that influence personal utility.”^34^ These directly map to themes we identified among the positive result population, indicating that these findings are consistent in 2 different populations. In the present study, however, we also interviewed parents of children who received negative and VUS results. Of note, these parents reported continued uncertainty about next steps in their child’s care in an already lengthy therapeutic journey. This directly opposes Jeffrey et al’s “Relief to end the diagnostic journey,” suggesting an important difference in perceptions between parents who receive a positive result and those that do not. Nonetheless, it is important to note that in this study, many of these parents still felt hopeful that future analysis could uncover additional causative variants.

### Limitations

Although we aimed to select participants with a wide variety of experiences with genetic testing, including those receiving negative, positive, and VUS results, recruitment was nonrandom and limited to a small sample size at a single center in western Canada. There was a gender imbalance in the study sample as respondents were predominantly mothers. Race and ethnicity demographics were not collected. Overall, participants were well educated and had received pre- and posttest genetic testing counseling. Results may be transferable to other populations but may not be generalizable. Further research can address these limitations by engaging broader communities both across Canada and globally.

On average, more than 4 years had passed between receiving genetic testing results and participating in this study, and therefore recall bias may have confounded findings as parents were providing retrospective accounts of their experience. Given that caregivers had previously agreed to participate in research, they may have had a more positive experience with genetic testing than those who declined to participate, contributing to an ascertainment bias. Additionally, hope was a common theme, being at least in part derived from the caregivers’ participation in this project as a genetic research endeavor. Accordingly, caregivers pursuing genetic testing as a purely clinical process may not share this experience. Furthermore, this study overrepresented parents of older children who had been searching for a diagnosis for many years. Further research is needed to capture the impact of an infant’s genetic diagnosis at an early age through whole exome sequencing or a similar next generation sequencing method as related results may yield a different experience than for families who have already had lengthy diagnostic journeys. As next generation sequencing becomes more integrated in the early phases of epilepsy care, these experiences will become increasingly important to consider. Families where both caregivers were interviewed provided an opportunity to compare experiences between caregivers of the same child, but they may have also shared similar experiences potentially skewing the data.

In this study, the methodology was developed based on the peer-reviewed literature of parent perceptions of genetic testing and molded around the interview results. This approach provided the necessary degree of flexibility to characterize novel themes. As discussed previously, however, the construct of personal utility defines well-established, standardized themes in epilepsy genetics that corresponded well to the themes found independently in this study. Accordingly, future studies may benefit from early integration of the personal utility construct,^35^ specifically in developing standard methods across epilepsy genetics research.

Finally, the study population contained only 2 children among the 23 from the original study by Demos et al whose management was altered as a direct result of the genetic diagnosis. It will be important to better capture the experiences of families who have changes to their treatment regimen in future studies as personalized medicine and gene therapies for epilepsy evolve.

## Conclusion

This study sought to understand the parent experience of navigating genetic testing for epilepsy. Major findings, in line with previous work, are as follows:

- Parents are keen to pursue genetic testing for their child to obtain a prognosis, inform treatment decisions, engage with other families, and exercise autonomy. Even if no genetic diagnosis is reached, caregivers feel that early testing is warranted to inform their child’s diagnostic odyssey.
- Receiving genetic results is an emotionally charged experience, and parents struggle with the uncertainty inherent in the process.
- Caregivers are hopeful that a genetic diagnosis will translate into specific treatments for their children.

As next generation sequencing technology is increasingly adopted into the workflow of epilepsy diagnosis and management, further longitudinal studies on parent perspectives in early-onset epilepsy are needed to understand how to support families and improve clinical outcomes.
